# The Role of Hydrotherapy in Enhancing Recovery After Knee Arthroplasty: A Systematic Review and Meta-Analysis of Randomized Controlled Trials

**DOI:** 10.3390/healthcare14132005

**Published:** 2026-07-06

**Authors:** Saja Nashmi Alrashedi, Eslam K. Fahmy, Hadaya Mosaad Eladl, Maha Ata Alshammari, Safya E. Esmaeel, Mustafa Shukry, Olfat Ibrahim Ali, Mohamed Abdelaziz Emam

**Affiliations:** 1 Department of Physical Therapy and Health Rehabilitation, College of Applied Medical Sciences, Jouf University, Sakaka 72388, Saudi Arabia; sn.alrashedii@gmail.com (S.N.A.); hd.mos@ju.edu.sa (H.M.E.); 2 Department of Physiology, College of Medicine, Northern Border University, Arar 91431, Saudi Arabia; eslam.kamal.fahmy@gmail.com (E.K.F.); safya.ebraheem@nbu.edu.sa (S.E.E.); 3 Department of Physical Therapy for Surgery, Faculty of Physical Therapy, Cairo University, Giza 12613, Egypt; 4 Department of Physical Therapy and Rehabilitation, Aljouf Cardiac Center, King Abdulaziz Specialist Hospital, Aljouf Health Cluster, Ministry of Health, Sakaka 72388, Saudi Arabia; 2260700012@iau.edu.sa; 5 Department of Biomedical Sciences, College of Veterinary Medicine, King Faisal University, P.O. Box 400, Al-Ahsa 31982, Saudi Arabia; matta@kfu.edu.sa; 6 Physical Therapy Program, Batterjee Medical College, Jeddah 21442, Saudi Arabia; 7 Department of Basic Science for Physical Therapy, Faculty of Physical Therapy, Cairo University, Giza 12613, Egypt; 8 János Szentágothai Neurosciences Division, Semmelweis University, 1085 Budapest, Hungary; 9 Basic Sciences Department, Faculty of Physical Therapy, Kafrelsheikh University, Kafr El-Sheikh 33511, Egypt

**Keywords:** hydrotherapy, total knee arthroplasty, postoperative pain, functional performance, muscle strength, aquatic therapy, systematic review, meta-analysis

## Abstract

Background: Total knee arthroplasty (TKA) is a common procedure to relieve pain and restore function in osteoarthritis patients. Postoperative rehabilitation is essential to address pain, swelling, reduced range of motion, and functional limitations. Hydrotherapy, using water buoyancy and resistance, may enhance recovery, but evidence on its effectiveness after these surgeries remains limited. Methods: A systematic literature search was conducted across six databases: PubMed, ProQuest, Science Direct, Google Scholar, Scopus, the Cochrane Library, and PEDro, covering studies published up to 30 November 2025. Only prospective randomized controlled trials were considered for inclusion. Studies such as case reports, uncontrolled case series, and those focused on outcomes other than postoperative pain and decreased muscle strength in patients undergoing total knee arthroplasty were excluded. This review was registered in PROSPERO (CRD420251164054). Results: Pooled analysis showed no statistically significant difference between hydrotherapy and land-based or usual-care rehabilitation in Visual Analogue Scale (VAS)-measured pain (MD ≈ −0.35; 95% CI [−1.06, 0.36]; p=0.34) or in WOMAC pain (MD ≈ −0.46; 95% CI [−8.50, 7.58]; p=0.89). In contrast, hydrotherapy produced a moderate, statistically significant improvement in lower-limb muscle strength (Hedges’ g=0.46; 95% CI [0.23, 0.69]), particularly in knee extensor and hip abductor strength. Heterogeneity was low for VAS pain and muscle strength but substantial for WOMAC pain ($I^{2}\approx 71\%$), and no evidence of publication bias was identified. Conclusions: Hydrotherapy did not reduce postoperative pain more than land-based exercise or usual care; pain relief was comparable between approaches, whereas hydrotherapy yielded greater gains in muscle strength. Heterogeneity in treatment parameters and the limited number of high-quality trials preclude definitive conclusions; future research should standardize hydrotherapy protocols and investigate long-term outcomes.

## 1. Introduction

Total knee arthroplasty (TKA) is widely utilized as a surgical intervention for managing end-stage osteoarthritis and other degenerative joint conditions [1], with the primary objectives of alleviating pain and enhancing joint function. Although these procedures are generally associated with favorable long-term outcomes, the immediate postoperative period is frequently characterized by substantial pain, edema, restricted range of motion (ROM), and impaired functional capacity [2]. These complications can significantly delay rehabilitation progress and extend hospital stays, thereby illustrating the clinical need for effective strategies to optimize early postoperative recovery and functional restoration [3].

Conventional postoperative rehabilitation following total knee arthroplasty typically begins within 24 h post-surgery. It emphasizes early mobilization, range-of-motion (ROM) exercises, progressive muscle strengthening, gait training, and functional task reconditioning. In TKA, early physical therapy includes bed mobility, ambulation with an assistive device, and passive to active ROM exercises performed to enhance functional recovery and reduce postoperative complications [4,5].

After surgery, a patient’s ability to engage in weight-bearing exercises may be restricted due to knee joint pain, swelling, or existing health conditions. Using an exercise environment that reduces pressure on the operated knee can help patients exercise more comfortably and effectively. Water, with its natural buoyancy and hydrostatic pressure, offers such an environment, making it a potentially beneficial option for rehabilitation following TKA [6]. Additionally, changes in the autonomic and circulatory systems when immersed in thermo-neutral water (34.5 °C) increase blood flow to muscles and tissues, aiding healing. Furthermore, the sensory interpretation of pain can also be modulated [7].

Aquatic training has been well studied in older adults [8], in patients with knee osteoarthritis experienced favorable improvements in mobility, functional performance, muscle strength, and pain [9], and has also proved effective in other populations: stroke [10], spinal cord injury [11], lymphedema [12], Parkinson’s disease [13], and chronic low back pain [14].

Current evidence on hydrotherapy following TKA remains heterogeneous, characterized by substantial variability in intervention protocols (e.g., initiation timing, water temperature, frequency, and duration), comparator conditions, and outcome measures. Moreover, previous reviews have frequently included mixed populations, non-randomized designs, or broader osteoarthritis cohorts, thereby limiting the specificity and clinical applicability of their findings to postoperative TKA rehabilitation. Uncertainty also persists regarding the effectiveness and optimal timing of hydrotherapy during the early postoperative phase, a critical period marked by significant functional impairment and rehabilitation demand. Despite growing interest in aquatic interventions as a low-impact rehabilitation strategy, their added value over conventional land-based approaches remains unclear. Recent evidence has continued to demonstrate the importance of rehabilitation interventions in improving pain, muscle strength, and functional outcomes following total knee arthroplasty, while emphasizing the need for high-quality randomized controlled trials and optimized rehabilitation protocols [15]. Accordingly, an updated systematic review and meta-analysis restricted to randomized controlled trials is warranted. This review aims to synthesize high-quality evidence, evaluate methodological rigor, and clarify the role of hydrotherapy in improving postoperative outcomes following TKA, thereby supporting evidence-based rehabilitation practice. Comparisons were made against land-based exercise or usual care. By evaluating the methodological quality and clinical relevance of existing trials, this review aims to clarify the therapeutic role of hydrotherapy in the postoperative management of major joint arthroplasty [14]. The novelty of the present review lies in its exclusive focus on randomized controlled trials evaluating hydrotherapy after TKA and its inclusion of the most recent evidence available up to November 2025, providing an updated and clinically relevant synthesis of its effectiveness in postoperative rehabilitation.

## 2. Materials and Methods

This review was prospectively registered in PROSPERO (ID: CRD420251164054). The protocol specified the eligibility criteria, search strategy, outcomes, and planned analyses.

### 2.1. Study Design

This systematic review and meta-analysis were conducted in accordance with PRISMA 2020 guidelines [16] to evaluate the effectiveness of hydrotherapy in improving postoperative outcomes among adults undergoing total knee arthroplasty.

### 2.2. Search Strategy

The electronic search was conducted across six databases (PubMed, ProQuest, ScienceDirect, Google Scholar, Scopus, the Cochrane Library, and PEDro) for studies published up to 30 November 2025. A comprehensive search strategy was developed using both controlled vocabulary (MeSH terms where applicable) and free-text keywords related to hydrotherapy and total knee arthroplasty. Search terms included combinations of: “hydrotherapy” OR “aquatic therapy” OR “water-based exercise” AND “knee arthroplasty” OR “total knee replacement” OR “TKA” AND “randomized controlled trial” OR “RCT”. Boolean operators (AND/OR) were used to combine search terms appropriately across databases. Database-specific filters for human studies and randomized controlled trials were applied where available. The full search strategy for each database is provided in Supplementary Material S1 to ensure reproducibility.

### 2.3. Eligibility Criteria

Studies were considered eligible if they were randomized controlled trials (RCTs) involving adult participants who had undergone total knee arthroplasty (TKA). Eligible interventions included hydrotherapy, aquatic physiotherapy, or aquatic resistance exercise, provided they were compared with land-based physiotherapy, usual care, or standard postoperative rehabilitation. Land-based exercise and usual care were selected as comparators as they reflect standard postoperative rehabilitation following TKA. Land-based therapy represented the conventional and most widely implemented approach, providing an appropriate reference for evaluating the comparative or additive effects of hydrotherapy. Usual care, encompassing basic mobilization and routine physiotherapy, reflected typical clinical practice and enables comparison with less intensive rehabilitation strategies. The inclusion of these comparators enhanced the clinical relevance and interpretability of the findings by contextualizing hydrotherapy within routine rehabilitation pathways and allowing evaluation of its role as either an adjunct or alternative to standard care. We would like to clarify that the included studies generally excluded patients with inflammatory or infectious joint conditions through their eligibility criteria. Examples of these exclusion criteria included rheumatoid arthritis, septic arthritis, postoperative deep joint infection, revision arthroplasty, and other major knee pathologies.

Studies were required to report at least one clinically relevant postoperative outcome—such as pain intensity (VAS or WOMAC pain subscale), muscle strength, or functional performance—and to provide sufficient quantitative data, including post-intervention means and standard deviations, to allow for inclusion in the meta-analysis. Exclusion criteria included non-randomized studies, observational research, quasi-experimental designs, or studies combining hydrotherapy with additional interventions that limit the ability to isolate the aquatic effect.

### 2.4. Study Selection

All search results were imported into the Rayyan platform (Rayyan Systems Inc., Cambridge, MA, USA), and duplicate records were removed. Two reviewers then independently screened titles and abstracts, followed by full-text evaluation of all studies deemed potentially eligible. Any discrepancies between reviewers were resolved through discussion or, when necessary, consultation with a third reviewer. A total of seven randomized controlled trials met the inclusion criteria, of which six were included in the final quantitative synthesis, as one study lacked extractable data required for meta-analysis.

### 2.5. Data Extraction

Data were independently extracted by two reviewers using a standardized extraction form. Extracted data included study characteristics, sample size, participant demographics, type of arthroplasty, intervention details (including frequency, duration, and intensity where reported), comparator characteristics, outcome measures, and post-intervention mean ± standard deviation values. Any discrepancies were resolved through consensus.

### 2.6. Risk of Bias Assessment

Risk of bias was assessed using the Cochrane Risk of Bias 2 tool (RoB 2.0) [17]. Each study was evaluated across the five domains and assigned an overall judgment of “low risk,” “some concerns,” or “high risk.”

### 2.7. Statistical Analysis

Statistical analyses were conducted in *R* (version 4.4.1; R Foundation for Statistical Computing, Vienna, Austria) using the meta and metafor packages. For WOMAC and VAS pain outcomes, pooled effects were calculated as mean differences (MDs) with 95% confidence intervals (CIs), as all included studies used identical measurement scales. Random-effects models were fitted using restricted maximum likelihood (REML) estimation with Hartung–Knapp adjustment.

Between-study heterogeneity was quantified using $I^{2}$ and $\tau^{2}$. For muscle strength outcomes with multiple correlated endpoints within studies (e.g., knee extension, knee flexion, hip abduction), robust variance estimation (RVE) within a random-effects framework was applied to account for within-study dependence. Publication bias was assessed using funnel plots, and Egger’s regression test was performed as an exploratory analysis given the small number of included studies.

## 3. Results

**Figure 1:**
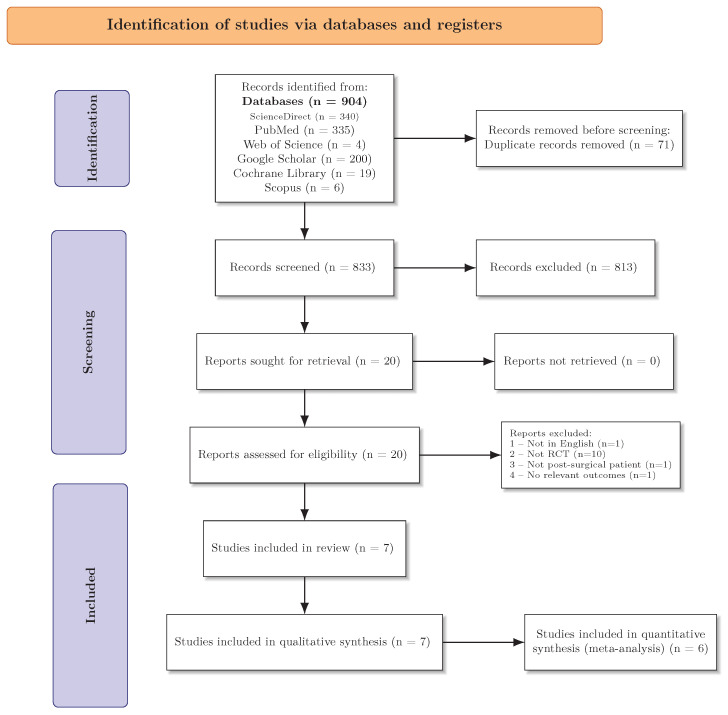
PRISMA 2020 flow diagram for the systematic literature search and study selection process.

The systematic search for this review identified aquatic rehabilitation trials following total knee arthroplasty. After screening and applying eligibility criteria, seven studies met the inclusion criteria, and six studies were incorporated into the final analysis after excluding one study because the data were not available (Figure 1).

Participants: Across the six included randomized controlled trials (RCTs), a total of 704 participants were enrolled. Sample sizes ranged from small pilot trials (20 participants) to large multicenter investigations (465 participants).

Interventions: A total of 6 RCTs were included for WOMAC pain, 3 for VAS pain, and 4 for different muscle strength outcomes (knee extension, flexion, and hip abduction measured in one study only). Besides the studies in the quantitative synthesis, one study by Giaquinto et al. [18] met the inclusion criteria but was not eligible for the meta-analysis because it lacked enough quantitative data (such as means and standard deviations). This study examined hydrotherapy after total knee arthroplasty and found significant improvements in pain, stiffness, and function using the WOMAC index [18]. The results favored hydrotherapy over conventional gym treatment, and the benefits were still present at a six-month follow-up. Accordingly, Giaquinto et al. [18] contributed to the qualitative (narrative) synthesis and, as part of the systematic review, was assessed for risk of bias; however, it was not included in the quantitative meta-analysis because it lacked extractable data. In total, seven RCTs met the inclusion criteria and underwent risk-of-bias assessment, of which six provided sufficient data for pooling. All studies compared hydrotherapy (either aquatic physiotherapy or aquatic resistance exercise) with land-based or usual care rehabilitation in adults after total knee replacement surgery. Most included populations were older adults (mean ages in the mid-60s to early 70s), and interventions were typically delivered during early post-operative recovery periods or shortly thereafter (Table 1).

### 3.1. Pain Outcomes: WOMAC Pain Subscale

A random-effects meta-analysis of six randomized controlled trials [6,19,20,21,22,23] compared hydrotherapy with control using WOMAC pain scores. As all studies used the same instrument, effects were synthesized as mean differences.

**Figure 2:**
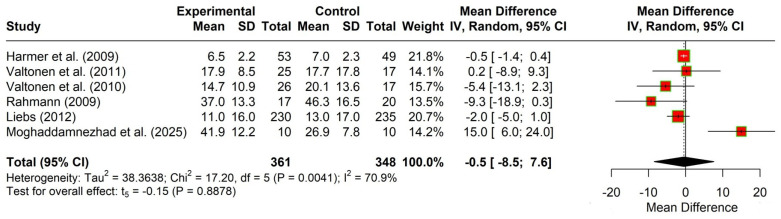
Forest plot showing the effect of interventions on pain as measured by the Western Ontario and McMaster Universities Osteoarthritis Index (WOMAC) Pain Subscale across included studies [6,19,20,21,22,23]. Mean differences (MDs) with 95% confidence intervals (CIs) are presented for each study and the overall pooled effect. Squares denote individual study estimates (sized by study weight), horizontal lines the 95% CIs, the diamond the pooled effect, and the dashed vertical line the line of no effect.

The pooled effect was not statistically significant (MD=−0.46, 95% CI [−8.50, 7.58], p=0.888) and indicated no clear between-group difference at post-intervention. Between-study heterogeneity was substantial ($I^{2}=70.9\%$, $\tau^{2}=38.36$), as shown in Figure 2.

### 3.2. Pain Outcomes: Visual Analogue Scale (VAS)

A random-effects meta-analysis of three randomized controlled trials [6,19,20] evaluated pain measured on the Visual Analogue Scale (VAS). As all studies used the same metric, results were pooled as mean differences.

**Figure 3:**
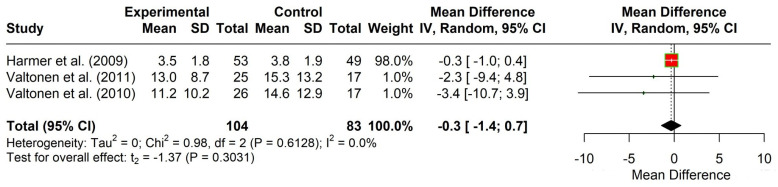
Forest plot depicting the impact of interventions on pain intensity measured using the Visual Analogue Scale (VAS) across the included studies [6,19,20]. Individual study effects and the overall pooled effect are shown with mean differences (MDs) and 95% confidence intervals (CIs). Squares denote individual study estimates (sized by study weight), horizontal lines the 95% CIs, the diamond the pooled effect, and the dashed vertical line the line of no effect.

The pooled estimate was not statistically significant (MD=−0.35, 95% CI [−1.06, 0.36], p=0.336). Statistical heterogeneity was low ($I^{2}=0\%$), although precision was limited by the small number of studies (Figure 3).

### 3.3. Muscle Strength Outcomes

A meta-analysis of 4 RCTs [6,19,20,21] examining muscle strength outcomes (knee extension, knee flexion, and hip abduction) was conducted using a random-effects model with robust variance estimation (RVE) to account for dependent effect sizes within studies. The pooled standardized mean difference indicated a moderate, statistically significant advantage for hydrotherapy (g=0.46; 95% CI [0.23, 0.69]). Study-specific SMDs included values from 0.26 to 1.16 for strength gains in favor of hydrotherapy, particularly for hip abduction and knee extension.

**Figure 4:**
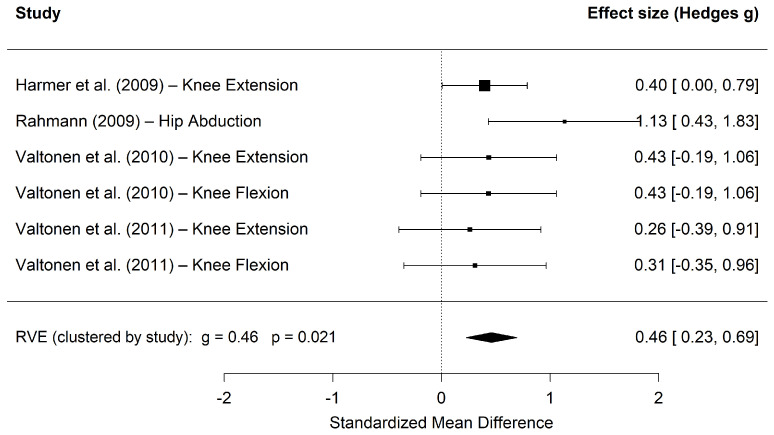
Forest plot showing the effect of interventions on muscle strength outcomes, including knee extension, knee flexion, and hip abduction, across included studies [6,19,20,21]. Effect sizes were estimated using Robust Variance Estimation (RVE). Squares denote individual study effect sizes (sized by study weight), horizontal lines the 95% CIs, the diamond the pooled effect, and the dashed vertical line the line of no effect.

There was minimal between-study heterogeneity ($\tau^{2}\approx 0$; $I^{2}=0\%$), and the overall effect remained statistically significant under RVE adjustment. Study-specific SMDs ranged from approximately 0.26 to 1.13, demonstrating consistent improvements in muscle strength, particularly for hip abduction and knee extension in favor of hydrotherapy (Figure 4).

### 3.4. Publication Bias

**Figure 5:**
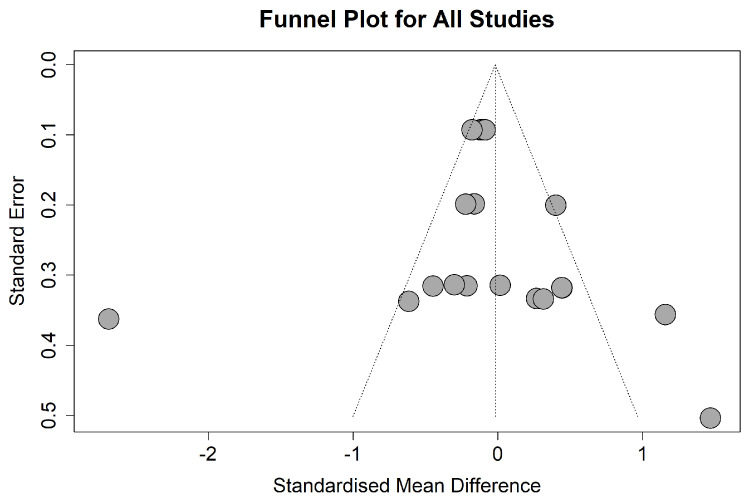
Funnel plot illustrating the assessment of publication bias among the included studies.

A funnel plot including all studies was constructed as an exploratory assessment of publication bias. Visual inspection of the funnel plot indicated no clear asymmetry. Egger’s linear regression test further supported the absence of small-study effects (t=0.81, df=18, p=0.43), with a non-significant intercept (bias estimate =0.78, SE=0.97). Residual heterogeneity remained substantial ($\tau^{2}=5.52$). Taken together, these findings suggest no evidence of publication bias. Given the relatively small number of included studies, these results should still be interpreted with caution (Figure 5).

### 3.5. GRADE Assessment

**Figure 6:**
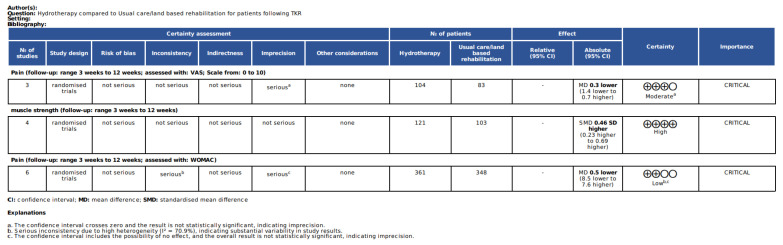
Summary of the GRADE evidence quality assessment for the included studies.

The quality of evidence and the strength of recommendations were assessed using the Grading of Recommendations Assessment, Development and Evaluation (GRADE) approach, which classifies evidence into four levels, high, moderate, low, and very low, based on factors such as risk of bias, inconsistency, indirectness, imprecision, and publication bias [24]. The results of the GRADE assessment are summarized in Figure 6.

### 3.6. Risk of Bias of Included Studies

**Figure 7:**
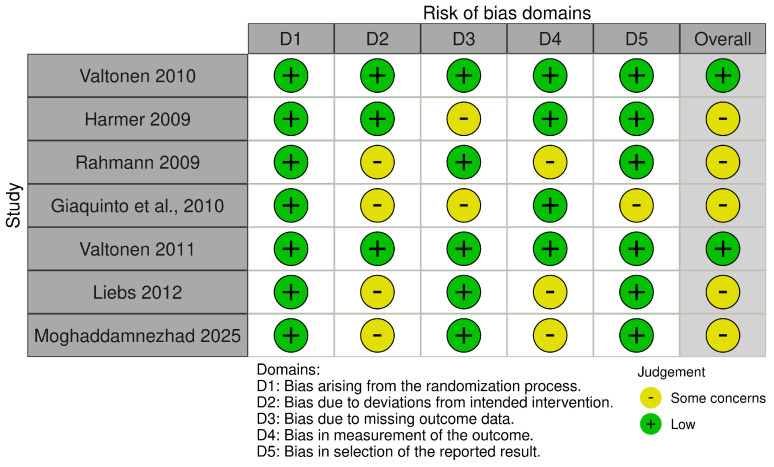
Summary of the risk of bias assessment for included studies presented using rounded, light-colored symbols (green = low risk, yellow = unclear risk) for each domain of the Cochrane Risk of Bias tool [6,18,19,20,21,22,23].

**Figure 8:**
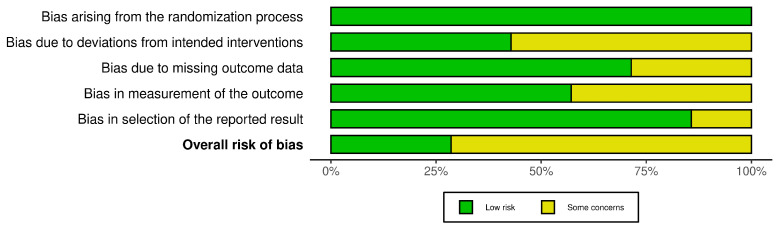
Summary of the risk of bias assessment for included studies presented as a horizontal bar chart.

Most of the included trials showed a low risk of bias across the five RoB 2 domains. Randomization and outcome measurement were consistently rated as low risk, indicating generally sound methodology. Some concerns were noted in missing outcome data and selective reporting, but these issues were limited and did not substantially weaken the evidence. Overall, the methodological quality was considered acceptable (Figure 7 and Figure 8).

## 4. Discussion

This systematic review and meta-analysis demonstrated that hydrotherapy following total knee arthroplasty (TKA) produced no statistically significant difference compared with land-based exercise or usual care in either VAS pain or WOMAC pain, but yielded a moderate, statistically significant improvement in muscle strength. These outcomes were derived from the included randomized controlled trials [6,18,19,21,22,23].

Although several individual trials reported reductions in pain following aquatic rehabilitation (Harmer et al. 2009 [6], Valtonen et al. 2010, 2011 [19,20], and Rahmann et al. 2009 [21]), the pooled analysis showed no statistically significant difference in VAS or WOMAC pain between hydrotherapy and land-based or usual-care rehabilitation. This indicates that hydrotherapy provided pain relief comparable to, rather than greater than, conventional land-based exercise. The moderate pooled gain in muscle strength also agrees well with the findings at an individual study level—in particular, the large gains in strength in the two Valtonen trials and the improvement in hip abduction strength described in Rahmann’s RCT [21]. These findings support the interpretation that buoyancy and hydrostatic pressure facilitate earlier activation, increased tolerance of exercise, and less joint compression, improving both pain-limited function and muscular recovery.

From a physiological perspective, these findings may be explained by the unique properties of the aquatic environment. Water buoyancy reduces effective body weight and mechanical loading on the operated knee joint, enabling patients to perform therapeutic exercises with less discomfort during the early postoperative period. In addition, hydrostatic pressure may facilitate venous return and reduce postoperative edema, thereby improving joint mobility and exercise tolerance. Warm-water immersion has also been associated with enhanced circulation, muscle relaxation, and modulation of pain perception. Collectively, these mechanisms may promote greater participation in rehabilitation and contribute to the observed improvements in muscle strength and to pain relief comparable with that achieved by land-based rehabilitation [7].

Despite multiple trials finding benefits, the total pooled improvement in WOMAC pain was not statistically significant. Specifically, Harmer [6], Valtonen [19,20], Rahmann [21], and Liebs [22] all observed reductions in WOMAC pain; however, variance in postoperative timing, length of intervention, and diverse research populations likely contributed to attenuation of the aggregate impact. WOMAC represents a multidimensional construct and may be less susceptible to short-term changes compared with VAS, which measures instantaneous pain intensity. The substantial statistical heterogeneity ($I^{2}\approx 71\%$) for this outcome, combined with residual clinical heterogeneity, suggests that differences in outcome measurement and timing may mask true population-level effects.

Comparison with previous evidence revealed findings that contrast with the present pooled results regarding pain outcomes. Alonso-Rodríguez et al. [25] reported significant improvements in pain, stiffness, gait performance, and muscle strength following hydrotherapy after TKA, which is consistent with the direction of the present findings. Similarly, Giaquinto et al. [18] demonstrated superior improvements in pain, stiffness, and physical function following hydrotherapy compared with conventional gym-based rehabilitation, with benefits maintained at six-month follow-up. These findings suggest that the clinical benefits of hydrotherapy may extend beyond the immediate postoperative period. Furthermore, evidence from a recent systematic review indicated that aquatic exercise is associated with significant reductions in pain and improvements in physical function among individuals with knee osteoarthritis [26]. These findings further contrast with the current pain results and may reflect differences in patient populations, intervention protocols, timing of treatment, and outcome measures between knee osteoarthritis and postoperative TKA rehabilitation settings.

The multicenter randomized controlled trial by Liebs et al. (2012) [22] reported joint-specific differences, demonstrating stronger effects in TKA, which may explain why WOMAC outcomes from trials combining total hip and knee arthroplasty did not reach statistical significance. These findings further highlight the influence of patient characteristics and intervention-related factors on the magnitude of treatment effects observed across studies.

The strengths of this review include the use of standardized effect sizes, random-effects modeling, formal assessment of publication bias, and the inclusion of both subjective outcome tools (VAS and WOMAC) and objective outcome measures (strength). The included trials ranged from small, controlled studies [23] to large multicenter cohorts [22], providing a wide evidence base. The funnel plot and Egger’s test did not reveal any significant publication bias.

The major limitations relate to heterogeneity of intervention across trials: different timing of treatment (early vs. late hydrotherapy), variable dosage, and program intensity. The interventions of Giaquinto [18] and Harmer [6] were shorter than the others, whereas protocols from Valtonen [19,20] involved extended resistance-based programs, thus adding to heterogeneity of effect size. Timing of WOMAC measurement varied significantly across studies and further explains the non-significant pooled effect. Variation in the observation period is a potential limitation of this systematic review. The follow-up durations of the included studies ranged from 2 to 24 weeks; future studies should comprise follow-up periods that are nearly similar. Furthermore, patient-related factors such as baseline functional status, habitual physical activity, obesity, comorbidities, adherence to rehabilitation, and lifestyle factors were not consistently reported across the included studies. These variables remain important sources of clinical heterogeneity that may have influenced postoperative recovery and treatment outcomes, and they could not be accounted for in the present synthesis given the nature of the available primary studies. We therefore recommend that future well-designed trials explicitly measure, report, and adjust for these factors so that their influence on the effectiveness of hydrotherapy can be more clearly determined.

Clinical interpretations of these findings indicated that hydrotherapy was a useful adjunct to standard postoperative rehabilitation. Rather than reducing pain more effectively than land-based exercise, hydrotherapy achieved comparable pain relief while producing greater gains in muscle strength and allowing rehabilitation with reduced mechanical loading on the operated joint. These properties make it a valuable alternative or complement to land-based rehabilitation, particularly when joint loading must be minimised. According to the evidence from the included studies and supportive external trials, hydrotherapy could be particularly beneficial during early-phase TKA rehabilitation. Future research should include joint-stratified RCTs, standardized hydrotherapy protocols, harmonized outcome sets, and appropriately powered multicenter studies to strengthen comparative estimates and identify optimum treatment parameters.

## 5. Conclusions

Hydrotherapy after total knee arthroplasty did not reduce postoperative pain more than land-based exercise or usual care: the pooled effects on both VAS and WOMAC pain were not statistically significant. Its principal benefit was a moderate, statistically significant improvement in muscle strength. Although several individual studies reported pain reductions, these did not translate into a significant pooled advantage, and the findings should be interpreted cautiously given the limited number of studies, the age of the evidence base, and differences in outcome measures, timing, and treatment protocols.

Token together, hydrotherapy may be considered a useful adjunct to standard postoperative rehabilitation. Further well-designed trials with standardized protocols and consistent outcome measures are needed to better define its role in pain reduction and optimize clinical use.

## Figures and Tables

**Table 1:** Summary of aquatic rehabilitation studies after knee arthroplasty.

Study	Participants	Intervention	Postoperative Interval	Observational Period	Frequency, Duration, and Water Temperature Used During Hydrotherapy Sessions	Duration and Frequency of Conventional Gym-Based Treatment	Main Findings
Harmer 2009 [6]	102 post-TKA patientsAge: Land 67.8 ± 6.3; Water 68.7 ± 9.1	Water-based vs. land-based rehabilitation	2 weeks post-surgery	24 weeks	2×/week, 60 min/session, for 6 weeks	Twice a week for 6 weeks; treatment sessions for each mode were 60 min long, which included 5-min warm-up and cool-down periods	Greater improvements in pain, WOMAC pain, and muscle strength in aquatic group.
Valtonen 2011 [19]	26 post-TKA patientsAge: 55–75	12-week aquatic resistance training	Unilateral knee replacement 4 to 18 months prior to the study	12 months	2×/week for 12 weeks	The control group did not receive any intervention	Reduced pain and WOMAC scores with large gains in knee muscle power.
Valtonen 2010 [20]	26 post-TKA patientsAge: 55–75	Aquatic resistance training	4 to 18 months prior to the study	12 weeks	2×/week for 12 weeks; 25–30 reps, 45 s work/30 s rest	The control group did not receive any intervention	Strength gains maintained at 12-month follow-up.
Rahmann 2009 [21]	65 post-TKA patientsAge: 69.6 ± 8.2	Aquatic physiotherapy	4 days postoperative	6 months	Daily until discharge; 10 reps/task; 34.5 °C water	For the 1st 3 days; ward exercise program (as per hospital’s clinical pathway)	Improved WOMAC pain and hip abduction strength.
Liebs 2012 [22]	465 post-TKA patientsAge: 66.3 ± 8.7	Aquatic physiotherapy	After 6 versus 14 days after TKA	24 months	30 min, 3×/week until postoperative week 5	30 min for 3 times a week up to the 5th postoperative week	Significant reduction in WOMAC pain.
Moghaddamnezhad 2025 [23]	20 post-TKA patientsAge: 52.4 ± 9.18	Aquatic physiotherapy (8 weeks)	One year had passed since the participants’ surgery	8 weeks	2×/week, 45–60 min/session for 8 weeks; 8–12 reps progression	The participants were massaged for 15 to 20 min in the massage therapy sessions (two sessions per week); each knee was massaged for eight weeks	Improved WOMAC scores, balance, TUG, and walking speed.
Giaquinto 2010 [18]	58 post-TKA patientsAge: ≈68	3-week hydrotherapy vs land-based therapy	Short interval from surgical intervention (less than 10 days)	6 months	40 min aquatic exercise after 20 min passive joint motion; 6×/week for 3 weeks	Same period of time; these received land therapy followed by a ‘neutral’ massage on the knee scar for 20 min; treatment sessions took place six times a week for three weeks	Greater improvements in pain and WOMAC outcomes.

## Data Availability

All data generated or analyzed during this study are included in this published article and its supplementary information files. Additional details are available from the corresponding author upon reasonable request.

## Supplementary Materials

The following supporting information can be downloaded at: https://www.mdpi.com/article/10.3390/healthcare14132005/s1.

## Abbreviations

The following abbreviations are used in this manuscript:

TKA	Total Knee Arthroplasty
ROM	Range of Motion
VAS	Visual Analogue Scale
WOMAC	Western Ontario and McMaster Universities Osteoarthritis Index
RCT	Randomized Controlled Trial
SMD	Standardized Mean Difference
MD	Mean Difference
CI	Confidence Interval
RVE	Robust Variance Estimation
REML	Restricted Maximum Likelihood
GRADE	Grading of Recommendations Assessment, Development and Evaluation
PRISMA	Preferred Reporting Items for Systematic Reviews and Meta-Analyses
