# Supplementary material for: The Role of Hydrotherapy in Enhancing Recovery After Knee Arthroplasty: A Systematic Review and Meta-Analysis of Randomized Controlled Trials
Source: Healthcare (Basel). 2026 Jul 6;14(13):2005. doi: 10.3390/healthcare14132005 (PMC13361691; doi:10.3390/healthcare14132005)
Supplement: Supplementary file 1 [file healthcare-14-02005-s001.zip › healthcare-4348870-supplementary.pdf]

| Database | Number of Records Identified | Search Syntax Used |
|---|---|---|
| ScienceDirect | n = 340 | "hydrotherapy" OR "aquatic therapy" OR "aqua therapy" OR "water-based exercise" AND "hip arthroplasty" OR "knee arthroplasty" AND "randomized controlled trial" OR "RCT" |
| PubMed | n = 335 | (hydrotherapy[Title/Abstract] OR aquatic therapy[Title/Abstract] OR aqua therapy[Title/Abstract] OR water-based exercise[Title/Abstract]) AND (hip arthroplasty[MeSH] OR knee arthroplasty[MeSH]) |
| Web of Science | n = 4 | TS=(hydrotherapy OR aquatic therapy OR aqua therapy OR water-based exercise) AND TS=(hip arthroplasty OR knee arthroplasty) AND TS=(randomized controlled trial OR RCT) |
| Google Scholar | n = 200 | "hydrotherapy" OR "aquatic therapy" OR "aqua therapy" OR "water-based exercise" "hip arthroplasty" OR "knee arthroplasty" "randomized controlled trial" OR "RCT" |
| Cochrane Library | n = 19 | hydrotherapy OR aquatic therapy OR aqua therapy OR water-based exercise AND hip arthroplasty OR knee arthroplasty |
| Scopus | n = 6 | TITLE-ABS-KEY("hydrotherapy" OR "aquatic therapy" OR "aqua therapy" OR "water-based exercise") AND TITLE-ABS-KEY("hip arthroplasty" OR "knee arthroplasty") AND TITLE-ABS-KEY("randomized controlled trial" OR RCT) |
| Total Records | n = 904 | |
